# Mutual solubilization of rosiglitazone and ibuprofen: Investigation and mechanistic insight

**DOI:** 10.1371/journal.pone.0345185

**Published:** 2026-03-18

**Authors:** Yaxiang Gong, Wenqi Liu

**Affiliations:** Department of Pharmacy, Linyi People’s Hospital, Linyi, China; University of Sahiwal, PAKISTAN

## Abstract

**Purpose:**

We report for the first time that co-dissolved rosiglitazone and ibuprofen mutually enhance their aqueous solubility; the study was designed to quantify this phenomenon and clarify its origin.

**Methods:**

Solubility was measured by HPLC. Fluorescence and FT-IR spectroscopy were used to probe molecular interactions, while classical molecular dynamics simulations provided atomistic insight.

**Results:**

The fluorescence intensity of the mixed solution was markedly lower than that of either drug alone, indicating ground-state complexation or close association. FT-IR spectra showed no new peaks, confirming the absence of a new chemical entity or distinct crystal phase. Simulations revealed that each drug increases the solvent–solute compatibility of the other; the resulting hetero-association hinders nucleation and crystal growth, raising apparent solubility. At 30–60 °C, rosiglitazone solubility rose from 60.4–79.9 to 69.2–92.4 μg/mL (+6–16%), while ibuprofen increased from 50.6–89.5 to 56.3–105.0 μg/mL (+11–27%), confirming mutual enhancement.

**Conclusion:**

The observed mutual solubilization is attributed to specific intermolecular interactions and enhanced solvent compatibility. These findings offer a simple, formulation-free strategy for improving the dissolution of poorly water-soluble drugs.

## Introduction

Diabetes mellitus is complex and chronic. Its prevalence is high and still rising [1–3]. Persistent hyperglycaemia causes multiple complications. These include cardiovascular disease, nephropathy, diabetic foot ulceration and retinopathy. Together they impose severe disability and economic hardship [4–7]. Accumulating evidence has established chronic, low-grade inflammation as a central driver of both metabolic dysregulation and end-organ injury [8–10]. Inflammatory mediators impair insulin signalling, intensify insulin resistance and thereby frustrate glycaemic control [11–13]; these same cytokines simultaneously orchestrate the microvascular and macrovascular sequelae of diabetes [14,15]. Adjunctive use of agents that suppress cytokine synthesis or signalling attenuates systemic inflammation, ameliorates insulin sensitivity, slows progression of complications and ultimately improves quality of life.

Contemporary clinical practice increasingly endorses rational polypharmacy for the management of multifactorial diseases [16–18]. Diabetic patients frequently require concomitant anti-inflammatory therapy for coexistent conditions such as osteoarthritis or cardiovascular disease [19]. Rosiglitazone, a thiazolidinedione insulin-sensitiser, activates peroxisome proliferator-activated receptor-γ, thereby augmenting insulin sensitivity in adipose and skeletal muscle tissue, improving glycaemic control and occupying an established niche in diabetes pharmacotherapy [20–22]. Ibuprofen, a widely prescribed non-steroidal anti-inflammatory drug, exerts potent antipyretic, analgesic and anti-inflammatory effects, and is routinely employed for the relief of mild-to-moderate pain and inflammatory states [23–25]. Its favourable risk-benefit profile underpins extensive worldwide utilization. The judicious co-prescription of rosiglitazone and ibuprofen thus carries tangible clinical utility: short-term ibuprofen affords symptomatic relief, enhances treatment adherence and preserves overall quality of life without compromising glycaemic management.

Rosiglitazone and ibuprofen are both extensively prescribed. However, their poor aqueous solubility constrains formulation development and limits systemic exposure [26–29]. Intrinsically low dissolution rates in the gastrointestinal milieu retard absorption, blunt maximum plasma concentrations and prolong the time to peak, thereby compromising rapid onset of action [30]. Moreover, restricted solubility can generate high local drug concentrations at the mucosal surface, increasing the likelihood of gastrointestinal irritation and dose-related adverse events [31].

To circumvent these liabilities, substantial research has been directed toward solubility enhancement through cocrystal engineering, solid-dispersion technologies and nano-strategies [32–34]. Representative examples include ibuprofen-nicotinamide cocrystal and rosiglitazone maleate salt, each of which demonstrably elevates apparent solubility and, consequently, oral bioavailability [35,36]. Although such approaches have yielded measurable gains, further optimization remains possible. Continued elucidation of the molecular mechanisms underpinning solubilization, coupled with the design of more efficient enabling technologies, is essential for maximising the therapeutic value of these otherwise poorly soluble agents.

Compared with the above-mentioned salt/co-crystal, solid-dispersion and nano-sizing tactics, the present strategy, mutual solubilisation between two poorly soluble drugs already in clinical use-has received only scant attention. Techniques such as melt extrusion, hydrotropy, mixed-solvent systems, micro-fluidic antisolvent precipitation and particle-size reduction have individually been applied to rosiglitazone or ibuprofen, yet they commonly demand additional excipients, complex equipment or energy-intensive steps. For rosiglitazone, cyclodextrin complexation, solid dispersions, and self-micro-emulsifying formulations have achieved solubility gains, while for ibuprofen, hydrotropic blends with niacinamide, melt-adsorbed silica systems and micro-fluidic-produced nano-suspensions have reported solubility improvements. The current work complements these efforts by demonstrating that simple co-loading of the two actives can raise their apparent aqueous solubility without any external excipient or processing aid, offering a formulation-sparing option particularly attractive for fixed-dose combination therapies.

## Materials and methods

### Materials

Rosiglitazone (98.5%) and ibuprofen (99.0%) were from Saen Chemical Technology (Shanghai, China). Acetonitrile was purchased from ANPEL Laboratory Technologies (Shanghai, China), and potassium bromide (99.0%) from Bide Pharmatech (Shanghai, China). Ultrapure water (18.2 MΩ·cm) was produced with a Milli-Q system (Millipore, USA).

### Solubility measurement

Excess rosiglitazone, ibuprofen, or an equimolar physical mixture of the two was added to 5 mL of distilled water and agitated for 48 h at 30, 40, 50 or 60 °C in a thermostatic bath(model 85−2, Shanghai Sile Instrument Co., Ltd.). Equilibrium was considered reached when undissolved solid remained visible by visual inspection and two 200-µL aliquots taken 6 h apart gave identical HPLC peak areas (±2%). After equilibration, aliquots were withdrawn, filtered through 0.22 µm PVDF membranes, and the filtrates analysed immediately by HPLC(Shimadzu Corporation, Japan). All solubility determinations were performed in triplicate (n = 3) on independently prepared suspensions. Values reported are the mean ± one standard deviation (SD). No outliers were detected and excluded.

Ibuprofen was separated on an Ultimate XB-C18 column (150 mm × 4.6 mm, 5 µm) at 40°C using a mobile phase of 40 mm phosphate buffer (pH 2.6)-acetonitrile (40:60, v/v) delivered at 0.8 mL min^-1^. Injection volume was 20 µL and detection wavelength 264 nm. The method was validated for linearity (R^2^ ≥ 0.99, 20–2000 µg/mL), accuracy (98–102% recovery) and precision (RSD < 2%, n = 6).

Rosiglitazone was analysed under identical flow and column conditions, but with phosphate buffer (pH 6.2)-acetonitrile (60:40, v/v) as eluent and UV detection at 247 nm. The method was validated for linearity (R^2^ ≥ 0.99, 20–200 µg/mL), accuracy (98–102% recovery) and precision (RSD < 2%, n = 6).

### Fluorescence analysis

Saturated aqueous solutions of rosiglitazone and ibuprofen were prepared separately at 25 °C; equal volumes (1:1, v/v) were then mixed to give the combination sample, while each drug was likewise diluted 1:1 with water to serve as individual controls. Emission was scanned from 300 to 400 nm with excitation at 295 nm using a multimode microplate reader(SpectraMax M5, Molecular Devices, USA), slit width 2 nm, scan speed 120 nm/min, path length 10 mm; solvent blanks were subtracted prior to data analysis.

### Fourier-transform infrared spectrophotometry

Fourier-transform infrared spectra were obtained on samples recovered from equilibrium solubility experiments. Potassium bromide was dried at 60 °C for 10 h. Excess drug was equilibrated in water at 70 °C; the resulting solution was slowly cooled to 25 °C and left open to the atmosphere to allow crystallization. The isolated solid was filtered, vacuum-dried and ground with KBr (1:50, w/w). Discs were pressed and scanned from 4000 to 400 cm^-1^ with 0.5 cm^-1^ resolution using a Fourier-transform infrared spectrometer (Nicolet iS5, Thermo Fisher Scientific, Waltham, MA, USA).

### Molecular simulation

Molecular simulations were performed with Materials Studio 7.0. The structures of rosiglitazone, ibuprofen and water were drawn, opened in Visualizer and energy-minimized using the Geometry Optimization task of the Forcite module with the Universal force field.

Solution boxes were constructed by packing the optimized solutes into a pre-equilibrated water cell. Each system was subjected to five annealing cycles (NVT, 308.15 to 500 to 308.15 K, 1 fs step, 100 ps per cycle) to remove local strain. Three compositions were prepared: (i) rosiglitazone + water, (ii) ibuprofen + water and (iii) rosiglitazone + ibuprofen + water.

Binding energies and solubility parameters were computed with the Blend module. Rosiglitazone was assigned as the base component and screened against (a) water and (b) ibuprofen + water; the procedure was repeated with ibuprofen as the base. All calculations employed the Universal force field for electrostatics.

Analogously, a ternary rosiglitazone-ibuprofen-water box was prepared through the same annealing protocol. Together with the previously generated binary rosiglitazone-water and ibuprofen-water cells, the three systems were analysed in the Forcite module to generate radial distribution functions. Production molecular dynamics (MD) were subsequently carried out in the NVT ensemble for 50 ns with a 1-fs time-step, long-range electrostatics were handled by the Ewald summation method, and van-der-Waals interactions were truncated at 12 Å. Frames were saved every 5 ps for computing radial distribution functions and ensemble averages. Three independent replicates were run for each composition to ensure statistical reliability.

## Results and discussion

### Solubility determination

**Fig 1:**
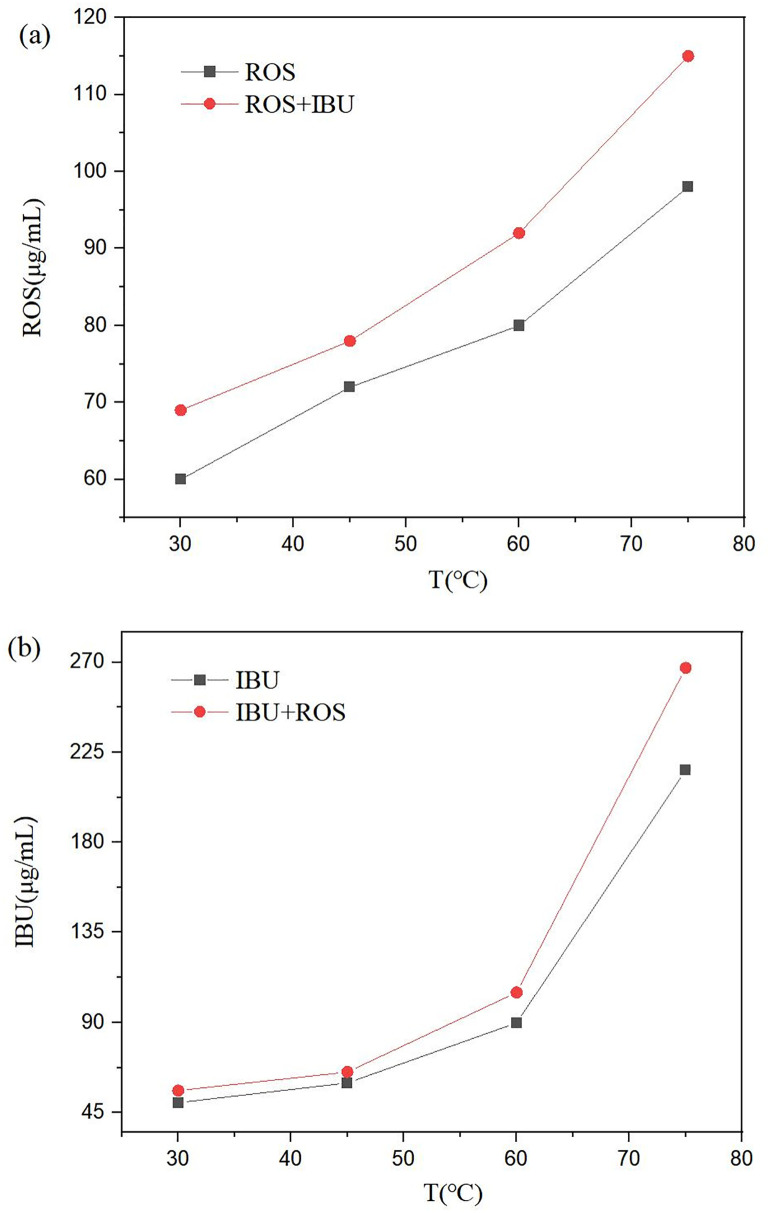
Solubility-temperature profiles: **(A)**, rosiglitazone in pure water and in rosiglitazone-water containing ibuprofen; **(B)**, ibuprofen in pure water and in ibuprofen–water containing rosiglitazone.

The solubility-temperature profiles (Fig 1) show a monotonic increase for both compounds. In Fig 1(a), the intrinsic solubility of rosiglitazone was 60.4 ± 4.2 μg/mL at 30 °C, 69.1 ± 2.2 μg/mL at 40 °C, 74.2 ± 2.2 μg/mL at 50 °C, and 79.9 ± 1.5 μg/mL at 60 °C. In the presence of ibuprofen, these values increased to 69.2 ± 0.4 μg/mL, 73.1 ± 0.6 μg/mL, 81.4 ± 4.3 μg/mL, and 92.4 ± 5.9 μg/mL, respectively, corresponding to enhancements of 14.6% (30 °C), 5.8% (40 °C), 9.7% (50 °C), and 15.6% (60 °C).

Fig 1(b) shows that the intrinsic solubility of ibuprofen was 50.6 ± 2.7 μg/mL at 30 °C, 55.7 ± 3.4 μg/mL at 40 °C, 63.4 ± 3.8 μg/mL at 50 °C, and 89.5 ± 8.1 μg/mL at 60 °C. When rosiglitazone was present, ibuprofen solubility rose to 56.3 ± 5.7 μg/mL, 61.7 ± 1.6 μg/mL, 80.3 ± 3.1 μg/mL, and 105.0 ± 1.8 μg/mL, respectively, representing increases of 11.3% (30 °C), 10.8% (40 °C), 26.7% (50 °C), and 17.3% (60 °C).

At 37 °C, the solubility of rosiglitazone alone was 67.1 ± 1.3 μg/mL, whereas it reached 70.2 ± 3.6 μg/mL in the presence of ibuprofen; similarly, ibuprofen alone exhibited a solubility of 55.3 ± 4.6 μg/mL, which increased to 58.8 ± 3.6 μg/mL when rosiglitazone was present. Additional measurements conducted in buffered media at pH 1.2, 4.5, and 6.8 (S1 File.Supplementary information of solubility) consistently demonstrated that the combined rosiglitazone-ibuprofen system afforded higher solubilities for both drugs relative to their individual counterparts.Throughout the 30–60 °C range, the concentration of each drug is always higher in the binary mixture than in its respective single-component solution, demonstrating a clear mutual solubilization between rosiglitazone and ibuprofen in aqueous media.

### Fluorescence analysis

**Fig 2:**
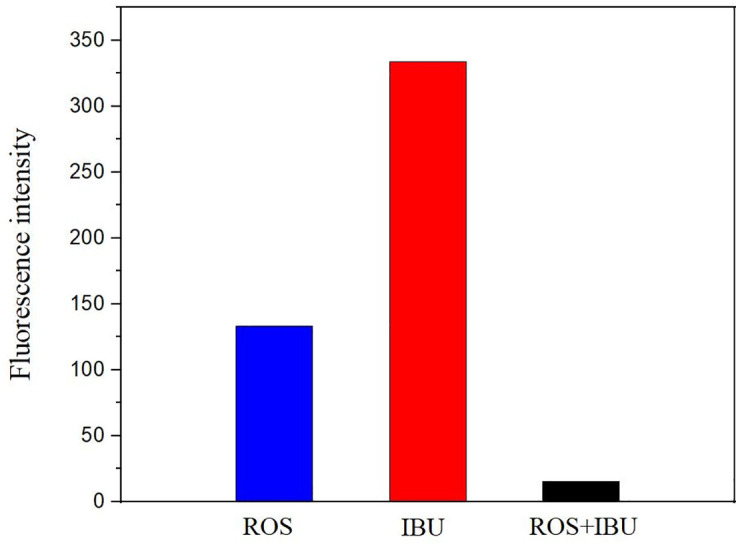
Fluorescence spectra of rosiglitazone, ibuprofen and their mixed solution.

Fluorescence quenching refers to the decrease in emission intensity when a fluorophore interacts with another species [37–39]. As shown in Fig 2, rosiglitazone and ibuprofen each exhibit strong emission at 320 nm. In the mixed solution, however, the intensity is lower than the sum of the individual signals and even below that of either drug alone, indicating ground-state association between the two molecules in aqueous medium.

**Fig 3:**
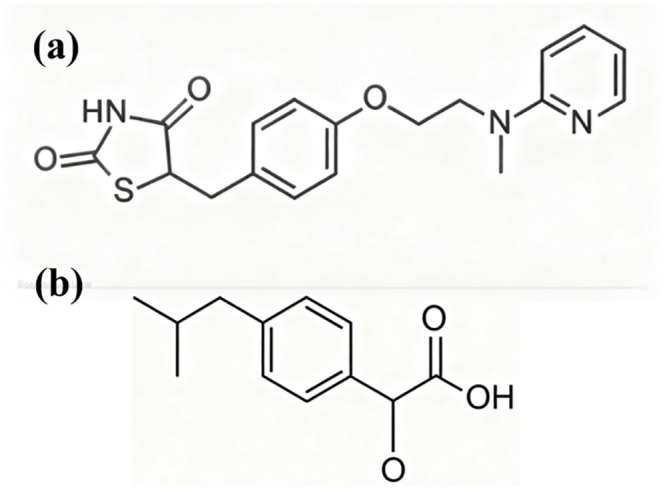
Chemical structure. **(A)**, rosiglitazone. **(B)**, ibuprofen.

Fluorescence quenching is commonly linked to direct intermolecular contact. Inspecting the structures (Fig 3) reveals that rosiglitazone contains both a phenyl and a pyridine ring, whereas ibuprofen bears a phenyl moiety; each possesses an extended π-electron system capable of light absorption and emission. When the two drugs approach in solution, these conjugated planes can engage through electrostatics, dipole-dipole coupling or π-π stacking, providing a structural basis for the observed loss of fluorescence intensity.

The nitrogen linking the pyridine and the oxygen attached to the phenyl in rosiglitazone, together with the carboxylate flanking the phenyl ring in ibuprofen, polarise the respective π-systems. When the two molecules approach, these local charges create an electrostatic field that perturbs the electron density of the opposite ring. In addition, both drugs possess extended conjugated moieties with permanent dipole moments; at short separations the dipoles align, forcing simultaneous rearrangement of π-electron clouds. The combined electrostatic and dipole-dipole interactions raise the energy of the excited π-state. They also provide non-radiative decay pathways. As a result, fluorescence is quenched. The observed loss of emission thus evidences direct molecular contact, a interaction that is plausibly responsible for the simultaneous increase in aqueous solubility. Fluorescence quenching can proceed through either dynamic (collisional) or static (ground-state complex) mechanisms. In the present study, the appearance of a single, uniformly diminished emission band without spectral shift or additional peaks, together with the absence of a measurable change in fluorescence lifetime (data not shown), supports a static process in which a non-fluorescent rosiglitazone-ibuprofen ground-state adduct is formed.

### Fourier-transform infrared spectrophotometry

**Fig 4:**
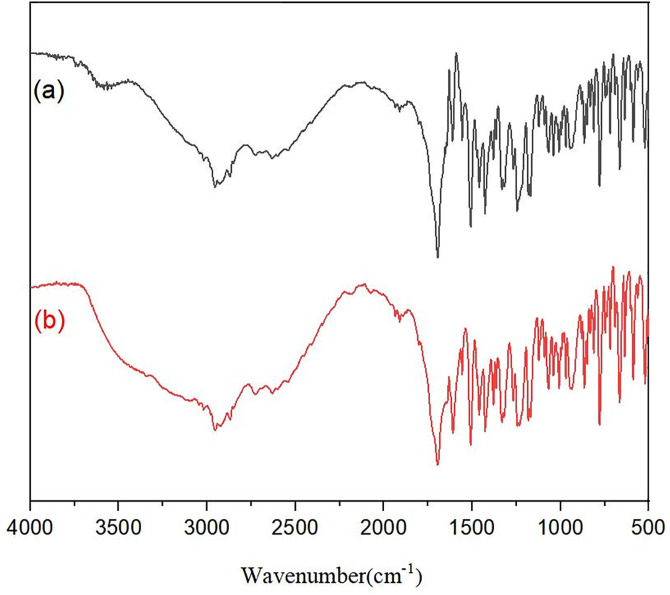
Fourier-transform infrared spectra. **(A)**, solid physical mixture of rosiglitazone and ibuprofen. **(B)**, crystalline material obtained from their aqueous co-solution.

Infrared analysis of the solid recovered from equilibrated rosiglitazone-ibuprofen solutions gave spectra superimposable on the physical mixture of the two pure drugs (Fig 4). No additional or shifted absorption bands appeared, confirming the absence of a new crystalline phase or covalent adduct; the precipitate consists simply of unchanged rosiglitazone and ibuprofen.

The FT-IR spectrum of the precipitate reproduced every major band of the physical mixture, 2955, 1696, 1608, 1500, 1420, 773, 663, 582 and 526 cm^-1^,without shift or loss (Fig 4). This one-to-one correspondence confirms that the functional groups of both drugs remain intact during crystallization, indicating that the aqueous interaction between rosiglitazone and ibuprofen does not generate a new chemical entity.

Crystallographic examination shows that rosiglitazone and ibuprofen retain their native bonding patterns when jointly present in water. Although their comparable pK_a_ values favour non-covalent association, successful cocrystal formation also demands geometric complementarity and precise supersaturation kinetics [40]. Under the conditions employed here, the intermolecular forces are evidently too weak to nucleate a distinct lattice; moreover, any solution-phase complex may be sufficiently soluble to resist isolation by simple precipitation. While the 0.5 cm^-1^ resolution used here reveals no discernible band shifts or new peaks, subtle alterations in hydrogen-bonding patterns below this detection limit cannot be completely ruled out; nevertheless, the overall evidence indicates that no new crystalline or covalent phase is formed under the tested conditions.

### Molecular simulation

**Fig 5:**
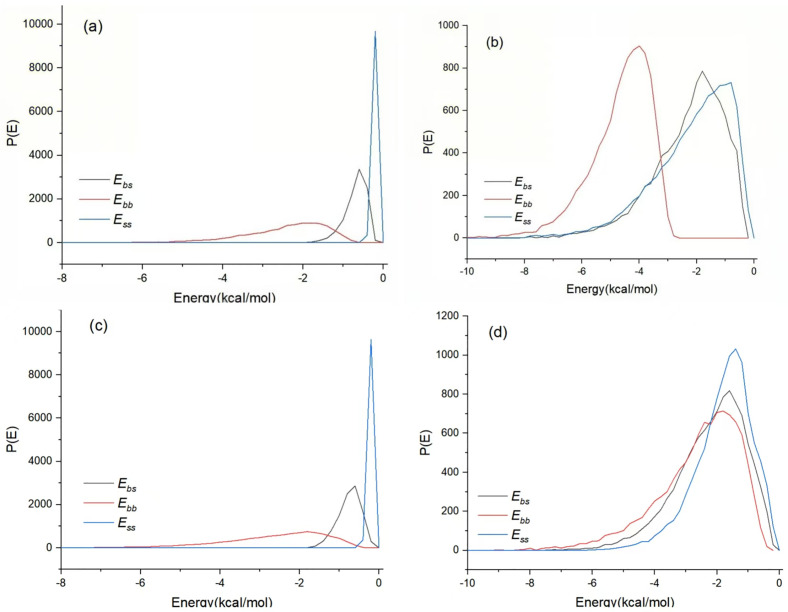
Distribution of solvent-solute binding energies under different systems. **(A)**, ibuprofen in pure water; **(B)**, ibuprofen in water containing rosiglitazone; **(C)**, rosiglitazone in pure water; **(D)**, rosiglitazone in water containing ibuprofen.

Molecular simulations were run to quantify the solvent compatibility of the two drugs. The interaction-energy distributions in Fig 5 show that, in the presence of ibuprofen, the rosiglitazone-water energy difference narrows markedly; conversely, rosiglitazone reduces the energy gap between ibuprofen and water. Thus each solute increases the thermodynamic likeness of the other to the aqueous phase, providing a plausible molecular origin for their mutual solubilization.

**Fig 6:**
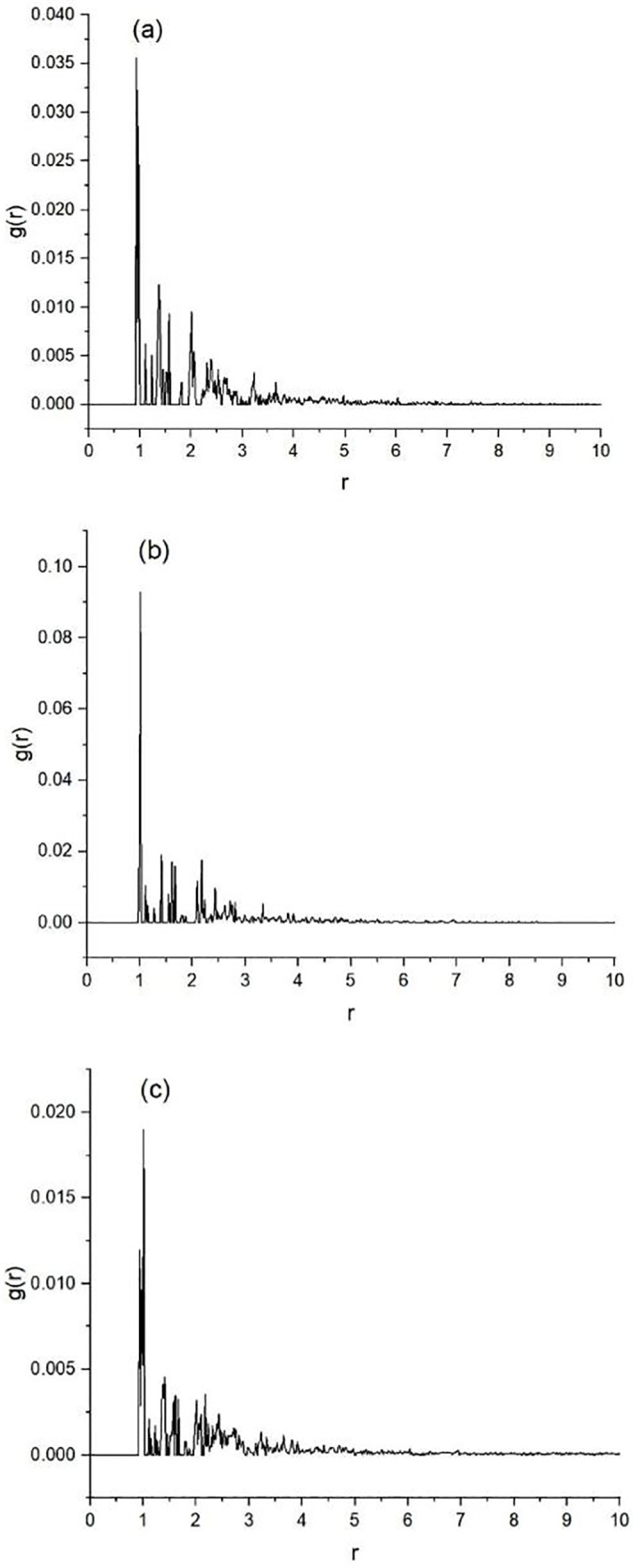
Radial distribution functions. **(A)**, rosiglitazone in water; **(B)**, ibuprofen in water; **(C)**, rosiglitazone and ibuprofen in mixed aqueous solution.

Radial distribution functions (Fig 6) exhibit the highest peak at ~1 Å for all systems, yet the amplitude is markedly lower in the mixed solution than in the single-drug analogues. The reduced peak height signals weaker solute-solute coordination and fewer productive encounters. Because nucleation and subsequent crystal growth require frequent and properly oriented collisions [41–43], the diminished correlation lengthens the induction period and elevates the apparent solubility of both compounds.

The simulations mirror the experimental observations and extend them to the molecular level. The measured solubility increase upon co-dissolution is accompanied by closer solute-solvent energetics and reduced solute-solute correlation, exactly the trends captured in the modelling. Thus the calculations corroborate the mutual solubilization detected in vitro and furnish a mechanistic rationale for it. It should be noted that the calculations treat aqueous media as pure water at fixed neutral pH; thus they do not capture the ionic strength, bile-micelle environment, or pH gradients encountered in the gastrointestinal tract, and the quantitative energetics may shift under physiological conditions.

Leveraging the observed mutual solubilisation in a dosage-form context could follow several straightforward paths. A simple co-solution fill-finish approach, for example, an immediate-release soft-gelatin capsule containing both drugs dissolved in a common, vehicle)would eliminate the need for salt or cocrystal screening while retaining rapid dissolution. Alternatively, co-granulation via wet or melt granulation, or co-precipitation from a shared solvent, could yield freely compressible granules with micro-environmental supersaturation that persists long enough to enhance absorption. For developers pursuing a cocrystal strategy, the present binding-energy data can guide stoichiometric selection and polymer-induced nucleation inhibition. Before clinical translation, standard pharmaceutical tests- dissolution under biorelevant conditions, physical stability (40 °C/75% RH, 6 months), and phase purity (PXRD, DSC) — should be performed; an in vivo pharmacokinetic bridging study in dogs or healthy volunteers would then confirm whether the in-vitro solubility gain translates into increased C_max_ or reduced T_max_ without altering overall exposure (AUC).

Although rosiglitazone and ibuprofen are often co-prescribed in real-world practice, this work addresses only their physicochemical solubility enhancement. Pharmacokinetic interactions or pharmacodynamic considerations (fluid retention, cardiovascular risk) remain outside the scope of the present study and must continue to guide clinical decision-making. Any future co-formulated product would therefore require dedicated bioequivalence and safety evaluation in the target population.

While the present work demonstrates increased aqueous solubility via hetero-association, heightened micellar or complex stability can, in some instances, reduce the free-drug fraction available for intestinal membrane permeation. Dedicated in-vitro transport experiments (for example, Caco-2 or PAMPA) and in-vivo pharmacokinetic studies are therefore required to verify that the solubility gain translates into faster absorption or higher bioavailability, rather than being offset by permeability limitations.

## Conclusion

Rosiglitazone and ibuprofen mutually enhance their aqueous solubility through direct molecular interactions that increase their compatibility with the solvent. This observation offers a simple, formulation-free strategy for improving the dissolution of poorly water-soluble drugs.

## Supporting information

S1 File Supplementary information of solubility. (DOCX)

S2 File Raw data. (XLSX)

S3 File Data of experiment. (RAR)

## References

[1] Winer N, Sowers JR. Epidemiology of diabetes. J Clin Pharmacol. 2004;44(4):397–405. doi: 10.1177/0091270004263017 15051748

[2] Roglic G. WHO Global report on diabetes: A summary. Int J Non-Commun Dis. 2016;1(1):3. doi: 10.4103/2468-8827.184853

[3] Zimmet PZ, Magliano DJ, Herman WH, Shaw JE. Diabetes: a 21st century challenge. Lancet Diabetes Endocrinol. 2014;2(1):56–64. doi: 10.1016/S2213-8587(13)70112-8 24622669

[4] Luo C, Zhang Y, Ding Y, Shan Z, Chen S, Yu M, et al. Nut consumption and risk of type 2 diabetes, cardiovascular disease, and all-cause mortality: a systematic review and meta-analysis. Am J Clin Nutr. 2014;100(1):256–69. doi: 10.3945/ajcn.113.076109 24847854

[5] Molitch ME, DeFronzo RA, Franz MJ, Keane WF, Mogensen CE, Parving H-H, et al. Nephropathy in diabetes. Diabetes Care. 2004;27 Suppl 1:S79-83. doi: 10.2337/diacare.27.2007.s79 14693934

[6] Nemcová J, Hlinková E, Martinická K. Patients’ experiences with diabetic foot ulceration -- a thematic analysis. Surg Vasc Nurs/ Pielegniarstwo Chir i Angiol. 2025;19.

[7] Kim S, Park J, Son Y, Lee H, Woo S, Lee M, et al. Development and Validation of a Machine Learning Algorithm for Predicting Diabetes Retinopathy in Patients With Type 2 Diabetes: Algorithm Development Study. JMIR Med Inform. 2025;13:e58107. doi: 10.2196/58107 39924304 PMC11830482

[8] Lontchi-Yimagou E, Sobngwi E, Matsha TE, Kengne AP. Diabetes mellitus and inflammation. Curr Diab Rep. 2013;13(3):435–44. doi: 10.1007/s11892-013-0375-y 23494755

[9] Wellen KE, Hotamisligil GS. Inflammation, stress, and diabetes. J Clin Invest. 2005;115(5):1111–9. doi: 10.1172/JCI25102 15864338 PMC1087185

[10] Tsalamandris S, Antonopoulos AS, Oikonomou E, Papamikroulis G-A, Vogiatzi G, Papaioannou S, et al. The Role of Inflammation in Diabetes: Current Concepts and Future Perspectives. Eur Cardiol. 2019;14(1):50–9. doi: 10.15420/ecr.2018.33.1 31131037 PMC6523054

[11] Greenfield JR, Campbell LV. Relationship between inflammation, insulin resistance and type 2 diabetes: “cause or effect”?. Curr Diabetes Rev. 2006;2(2):195–211. doi: 10.2174/157339906776818532 18220627

[12] Dandona P, Aljada A, Bandyopadhyay A. Inflammation: the link between insulin resistance, obesity and diabetes. Trends Immunol. 2004;25(1):4–7. doi: 10.1016/j.it.2003.10.013 14698276

[13] King GL. The role of inflammatory cytokines in diabetes and its complications. J Periodontol. 2008;79(8 Suppl):1527–34. doi: 10.1902/jop.2008.080246 18673007

[14] Nedosugova LV, Markina YV, Bochkareva LA, Kuzina IA, Petunina NA, Yudina IY, et al. Inflammatory Mechanisms of Diabetes and Its Vascular Complications. Biomedicines. 2022;10(5):1168. doi: 10.3390/biomedicines10051168 35625904 PMC9138517

[15] Soumya D, Srilatha B. Late Stage Complications of Diabetes and Insulin Resistance. J Diabetes Metab. 2011;02(09). doi: 10.4172/2155-6156.1000167

[16] Pollack RM, Donath MY, LeRoith D, Leibowitz G. Anti-inflammatory Agents in the Treatment of Diabetes and Its Vascular Complications. Diabetes Care. 2016;39 Suppl 2:S244-52. doi: 10.2337/dcS15-3015 27440839

[17] Bellucci PN, González Bagnes MF, Di Girolamo G, González CD. Potential Effects of Nonsteroidal Anti-Inflammatory Drugs in the Prevention and Treatment of Type 2 Diabetes Mellitus. J Pharm Pract. 2017;30(5):549–56. doi: 10.1177/0897190016649551 27194069

[18] Heerspink HJL, De Zeeuw D. Novel anti-inflammatory drugs for the treatment of diabetic kidney disease. Diabetologia. 2016;59(8):1621–3. doi: 10.1007/s00125-016-4030-4 27338625 PMC4930464

[19] Tabas I, Glass CK. Anti-inflammatory therapy in chronic disease: challenges and opportunities. Science. 2013;339(6116):166–72. doi: 10.1126/science.1230720 23307734 PMC3608517

[20] Xu B, Xing A, Li S. The forgotten type 2 diabetes mellitus medicine: rosiglitazone. Diabetol Int. 2021;13(1):49–65. doi: 10.1007/s13340-021-00519-0 35059243 PMC8733070

[21] Majidi Z, Hosseinkhani S, Amiri-Dashatan N, Emamgholipour S, Tutunchi S, Hashemi J, et al. Effect of rosiglitazone on circulating malondialdehyde (MDA) level in diabetes based on a systematic review and meta-analysis of eight clinical trials. J Investig Med. 2021;69(3):697–703. doi: 10.1136/jim-2020-001588 33408159

[22] Qu B, Zeng Z, Yang H, He J, Jiang T, Xu X, et al. Resveratrol reversed rosiglitazone administration induced bone loss in rats with type 2 diabetes mellitus. Biomed Pharmacother. 2024;178:117208. doi: 10.1016/j.biopha.2024.117208 39088966

[23] Ferreira BL, Ferreira DP, Borges SF, Ferreira AM, Holanda FH, Ucella-Filho JGM, et al. Diclofenac, ibuprofen, and paracetamol biodegradation: overconsumed non-steroidal anti-inflammatories drugs at COVID-19 pandemic. Front Microbiol. 2023;14:1207664. doi: 10.3389/fmicb.2023.1207664 37965564 PMC10642723

[24] Abbas AM, Aboelmagd A, Kishk SM, Nasrallah HH, Boyd WC, Kalil H, et al. A Novel Ibuprofen Derivative and Its Complexes: Physicochemical Characterization, DFT Modeling, Docking, In Vitro Anti-Inflammatory Studies, and DNA Interaction. Molecules. 2022;27(21):7540. doi: 10.3390/molecules27217540 36364366 PMC9653649

[25] Ngernyen Y, Petsri D, Sribanthao K, Kongpennit K, Pinijnam P, Pedsakul R, et al. Adsorption of the non-steroidal anti-inflammatory drug (ibuprofen) onto biochar and magnetic biochar prepared from chrysanthemum waste of the beverage industry. RSC Adv. 2023;13(21):14712–28. doi: 10.1039/d3ra01949g 37197677 PMC10184006

[26] Zhou X, Xiong A, Zhen Y, Yan Y, Zhang X. Solubility behavior of Rosiglitazone in ten pure solvents: Model correlation, thermodynamic calculations, and molecular simulation. J Mol Liq. 2025;417:126543.

[27] Sherje AP, Desai KJ. Spectrophotometric Determination of Poorly Water Soluble Drug Rosiglitazone Using Hydrotropic Solubilization technique. Indian J Pharm Sci. 2011;73(5):579–82. doi: 10.4103/0250-474X.99021 22923874 PMC3425073

[28] Irvine J, Afrose A, Islam N. Formulation and delivery strategies of ibuprofen: challenges and opportunities. Drug Dev Ind Pharm. 2018;44(2):173–83. doi: 10.1080/03639045.2017.1391838 29022772

[29] Faruki MZ, Razzaque E, Bhuiyan MA. Improvement of solubility of badly water soluble drug (Ibuprofen) by using surfactants and carriers. Int J Pharm Sci Res. 2013;4:1569.

[30] Ilevbare GA, Liu H, Edgar KJ, Taylor LS. Impact of polymers on crystal growth rate of structurally diverse compounds from aqueous solution. Mol Pharm. 2013;10(6):2381–93. doi: 10.1021/mp400029v 23597294

[31] Kadam SM, Kadam SR, Patil U, Ratan G, Jamkandi V. Review on floating drug delivery systems: an approach to oral controlled drug delivery via gastric retention. Int J Res Ayurveda Pharm. 2011.

[32] Hassan A, Khan JA, Nasir F, Shabir H, Hannan PA, Ullah R. Synthesis, characterization, and stability optimization of ibuprofen cocrystals employing various hydrophilic polymers. Curr Pharm Des. 2025;31:873–83. 38867533 10.2174/0113816128305926240530051853

[33] Biedrzycka K, Marcinkowska A. The Use of Hot Melt Extrusion to Prepare a Solid Dispersion of Ibuprofen in a Polymer Matrix. Polymers (Basel). 2023;15(13):2912. doi: 10.3390/polym15132912 37447557 PMC10346756

[34] Kumar N, Tyagi N, Mehan S, Singh AP. Formulation of Solid Lipid Nanoparticles Loaded with Rosiglitazone and Probiotic: Optimization and In-vitro Characterization. Recent Pat Nanotechnol. 2024;18(4):527–42. doi: 10.2174/0118722105268801231203144554 38305310

[35] Zhiming S, Chuang X, Wei D, Qiuxiang Y. Preparation of ibuprofen-nicotinamide cocrystal and its solubility measurement. Chem Ind Eng. 2014.

[36] Sorbera LA, Rabasseda X, Castañer J. Rosiglitazone maleate. Drugs Future. 1998;23:774.

[37] Siddiqui S, Ameen F, ur Rehman S, Sarwar T, Tabish M. Studying the interaction of drug/ligand with serum albumin. J Mol Liq. 2021;336:116200.

[38] Wani TA, Alanazi MM, Alsaif NA, Bakheit AH, Zargar S, Alsalami OM, et al. Interaction Characterization of a Tyrosine Kinase Inhibitor Erlotinib with a Model Transport Protein in the Presence of Quercetin: A Drug-Protein and Drug-Drug Interaction Investigation Using Multi-Spectroscopic and Computational Approaches. Molecules. 2022;27(4):1265. doi: 10.3390/molecules27041265 35209054 PMC8874853

[39] Sett R, Paul BK, Guchhait N. Deciphering the fluorescence quenching mechanism of a flavonoid drug following interaction with human hemoglobin. J Phys Org Chem. 2022;35:e4307.

[40] Khurshid A, Saeed A, Hökelek T, Taslim U, Irfan M, Khan SU, et al. Experimental and Hirshfeld Surface Investigations for Unexpected Aminophenazone Cocrystal Formation under Thiourea Reaction Conditions via Possible Enamine Assisted Rearrangement. Crystals. 2022;12(5):608. doi: 10.3390/cryst12050608

[41] Li J, Deepak FL. In Situ Kinetic Observations on Crystal Nucleation and Growth. Chem Rev. 2022;122(23):16911–82. doi: 10.1021/acs.chemrev.1c01067 36347015

[42] Gebauer D, Gale JD, Cölfen H. Crystal Nucleation and Growth of Inorganic Ionic Materials from Aqueous Solution: Selected Recent Developments, and Implications. Small. 2022;18(28):e2107735. doi: 10.1002/smll.202107735 35678091

[43] Xu S, Cao D, Liu Y, Wang Y. Role of Additives in Crystal Nucleation from Solutions: A Review. Crystal Growth & Design. 2021;22(3):2001–22. doi: 10.1021/acs.cgd.1c00776

