# Supplementary material for: Mutual solubilization of rosiglitazone and ibuprofen: Investigation and mechanistic insight
Source: PLoS One. 2026 Mar 18;21(3):e0345185. doi: 10.1371/journal.pone.0345185 (PMC12998801; doi:10.1371/journal.pone.0345185)

Supporting Information

**Mutual Solubilization of Rosiglitazone and Ibuprofen: Investigation and Mechanistic Insight**

Yaxiang Gong^a,^*, Wenqi Liu^a^,

^a^Department of Pharmacy, Linyi People’s Hospital, Linyi 276003, Chinaa

Corresponding authors:

Dr. Yaxiang Gong

Department of Pharmacy, Linyi People’s Hospital

Tel.: +86 15251756732, Email address: Arnold_bear@126.com

**Materials and methods.**

**Experimental section.**

**Solubility measurement**

Excess rosiglitazone, ibuprofen, or an equimolar physical mixture of the two was added to 5 mL of phosphate buffer(pH=1.2, 4.5, 6.8) and agitated for 48 h at 30, 40, 60 50 or 60°C in a thermostatic bath(model 85-2, Shanghai Sile Instrument Co., Ltd.). After equilibration, aliquots were withdrawn, filtered through 0.22 µm PVDF membranes, and the filtrates analysed immediately by HPLC(Shimadzu Corporation, Japan).

Ibuprofen was separated on an Ultimate XB-C18 column (150 mm × 4.6 mm, 5 µm) at 40°C using a mobile phase of 40 mm phosphate buffer (pH 2.6)-acetonitrile (40:60, v/v) delivered at 0.8 mL min-1. Injection volume was 20 µL and detection wavelength 264 nm.

Rosiglitazone was analysed under identical flow and column conditions, but with phosphate buffer (pH 6.2)-acetonitrile (60:40, v/v) as eluent and UV detection at 247 nm.

.

**Results and discussion**

Solubility measurements of rosiglitazone and ibuprofen were conducted in buffered media at pH 1.2, 4.5, and 6.8. At pH 1.2, the intrinsic solubility of rosiglitazone was 55.4μg/mL at 30°C, 64.6μg/mL at 40°C, 70.1μg/mL at 50°C, and 75.5μg/mL at 60°C. In the presence of ibuprofen, these values increased to 64.2μg/mL, 69.8μg/mL, 75.9μg/mL, and 88.4μg/mL, respectively, corresponding to enhancements of 15.9 % (30°C), 8.0 % (40°C), 8.3 % (50°C), and 16.6 % (60°C). Under the same conditions, ibuprofen alone exhibited solubilities of 48.2μg/mL, 54.6μg/mL, 61.3μg/mL, and 84.1μg/mL, which rose to 50.1μg/mL, 57.9μg/mL, 75.1μg/mL, and 94.4μg/mL when rosiglitazone was present, representing increases of 3.9 %, 6.0 %, 22.5 %, and 11.8 %.

At pH 4.5, rosiglitazone solubility was 61.8μg/mL at 30°C, 71.4μg/mL at 40°C, 77.1μg/mL at 50°C, and 83.4μg/mL at 60°C; co-dissolved ibuprofen raised these values to 72.3μg/mL, 76.1μg/mL, 83.6μg/mL, and 95.9μg/mL, gains of 17.0 %, 6.6 %, 8.4 %, and 15.0 %. Ibuprofen alone dissolved to 51.4μg/mL, 58.9μg/mL, 66.7μg/mL, and 96.5μg/mL, whereas in the presence of rosiglitazone it reached 59.7μg/mL, 65.6μg/mL, 83.9μg/mL, and 95.9μg/mL, reflecting increases of 16.1 %, 11.4 %, 25.8 %, and 15.0 %.

At pH 6.8, intrinsic rosiglitazone solubilities were 69.3μg/mL, 75.6μg/mL, 79.8μg/mL, and 86.6μg/mL at 30°C, 40°C, 50°C, and 60°C, respectively; with ibuprofen they rose to 74.9μg/mL, 79.3μg/mL, 86.2μg/mL, and 98.9μg/mL, corresponding to 8.1 %, 4.9 %, 8.0 %, and 14.2 % enhancement. Ibuprofen alone was markedly more soluble at this pH, giving 1030.2μg/mL, 1085.7μg/mL, 1293.0μg/mL, and 1423.5μg/mL; co-dissolved rosiglitazone further increased these values to 1090.0μg/mL, 1165.3μg/mL, 1357.9μg/mL, and 1499.3μg/mL, reflecting increases of 2.8 %, 7.4 %, 4.9 %, and 5.5 %, respectively.

**Legend of supplementary figures**

**Figure S1** Solubility temperature profiles: rosiglitazone and rosiglitazone containing ibuprofen at pH 1.2.


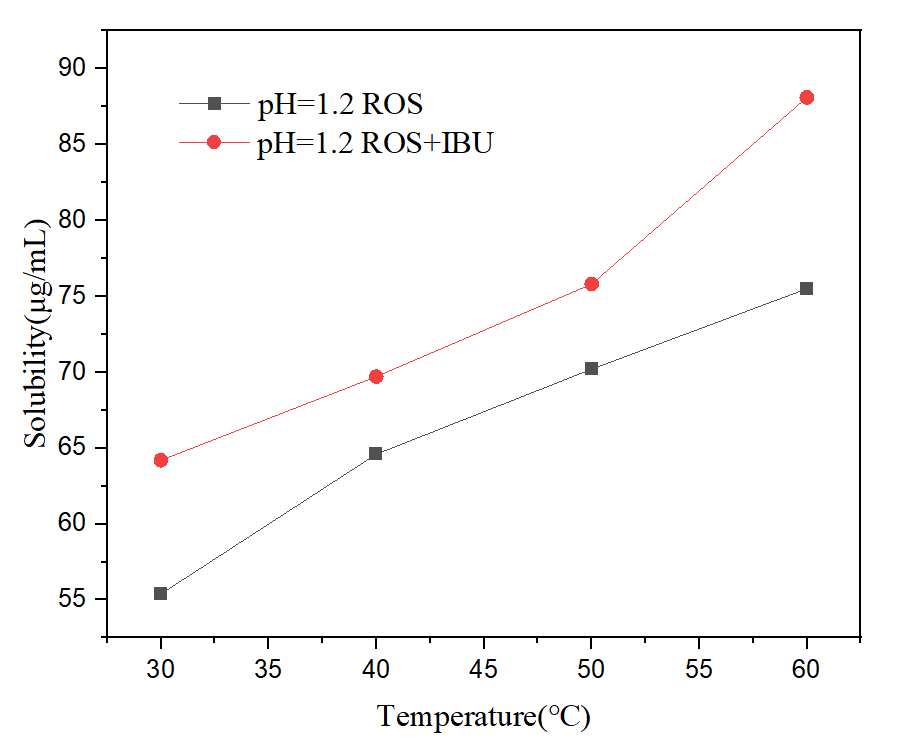


**Figure S2** Solubility temperature profiles: rosiglitazone and rosiglitazone containing ibuprofen at pH 4.5.


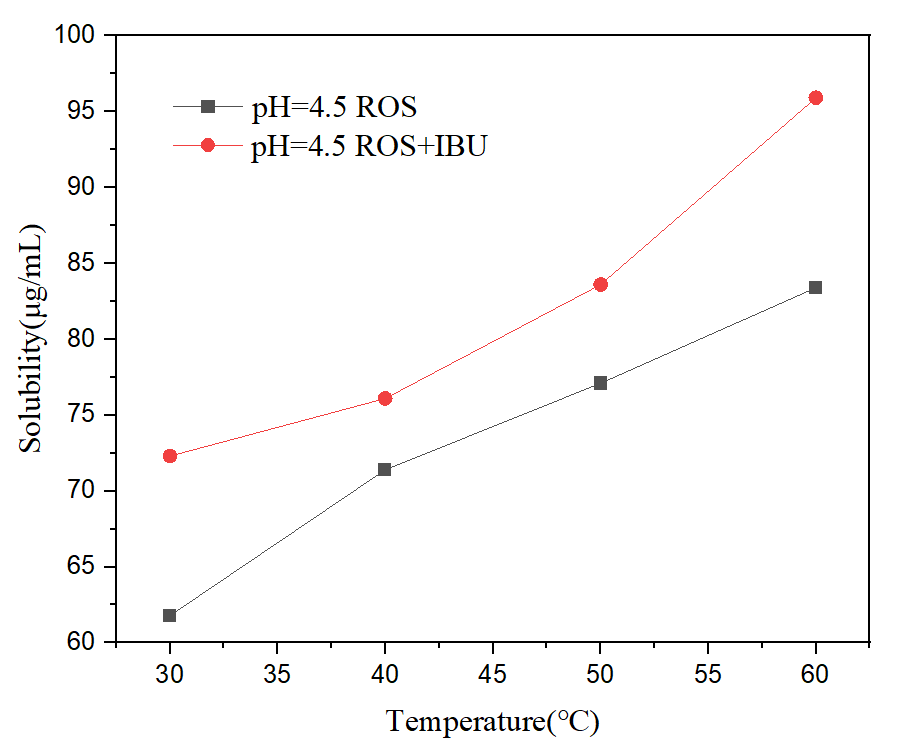


**Figure S3** Solubility temperature profiles: rosiglitazone and rosiglitazone containing ibuprofen at pH 6.8.


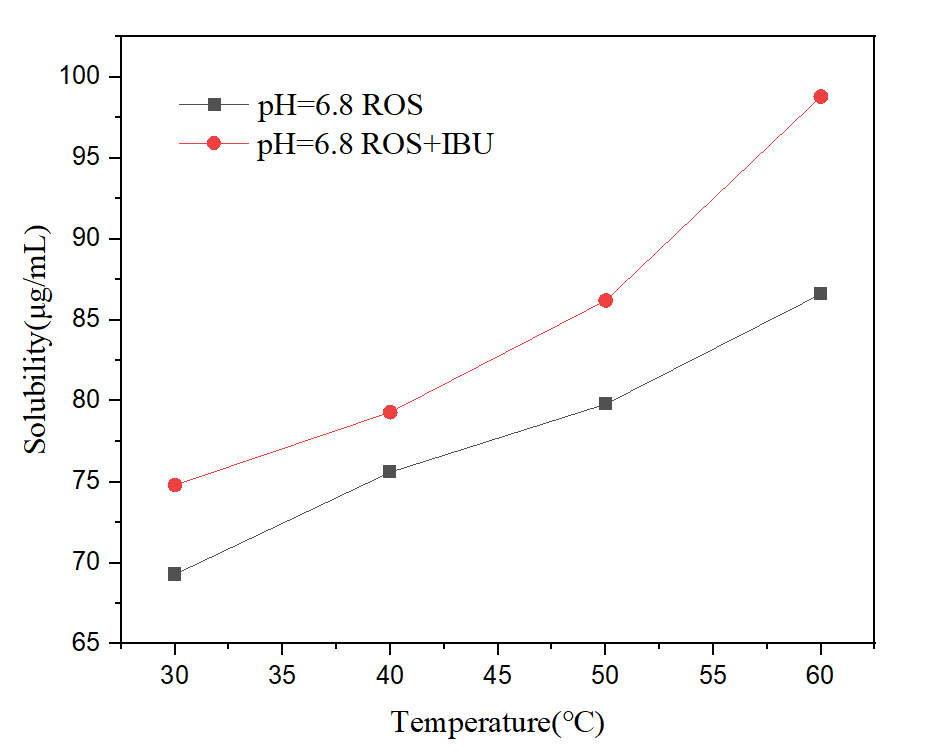


**Figure S4** Solubility temperature profiles: ibuprofen and ibuprofen containing rosiglitazone at pH 1.2.


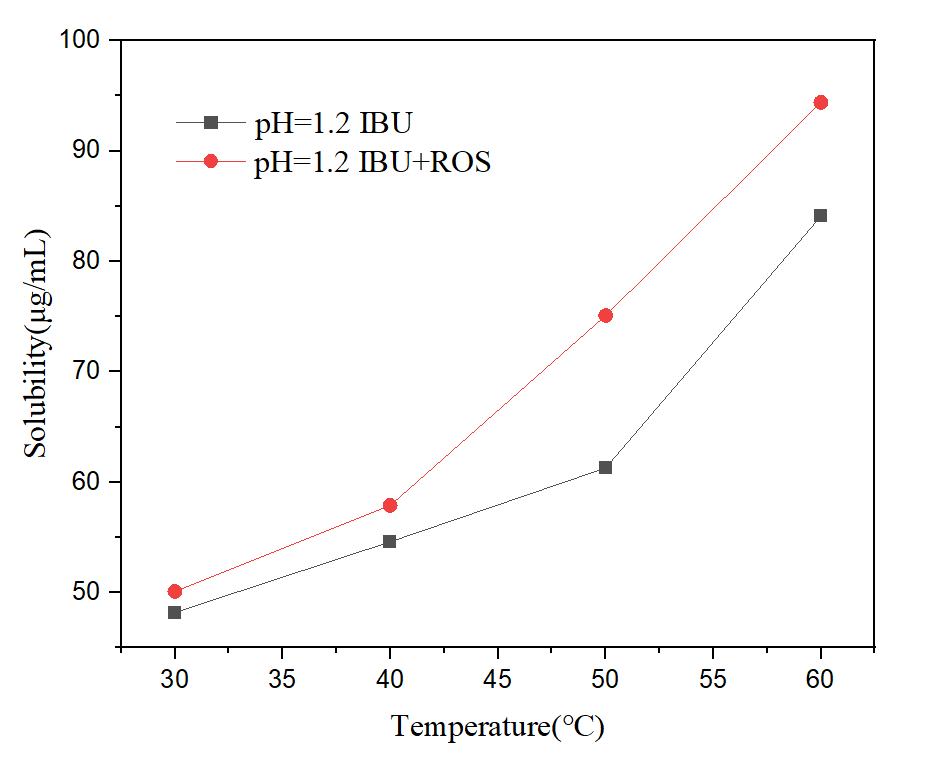


**Figure S5** Solubility temperature profiles: ibuprofen and ibuprofen containing rosiglitazone at pH 4.5.


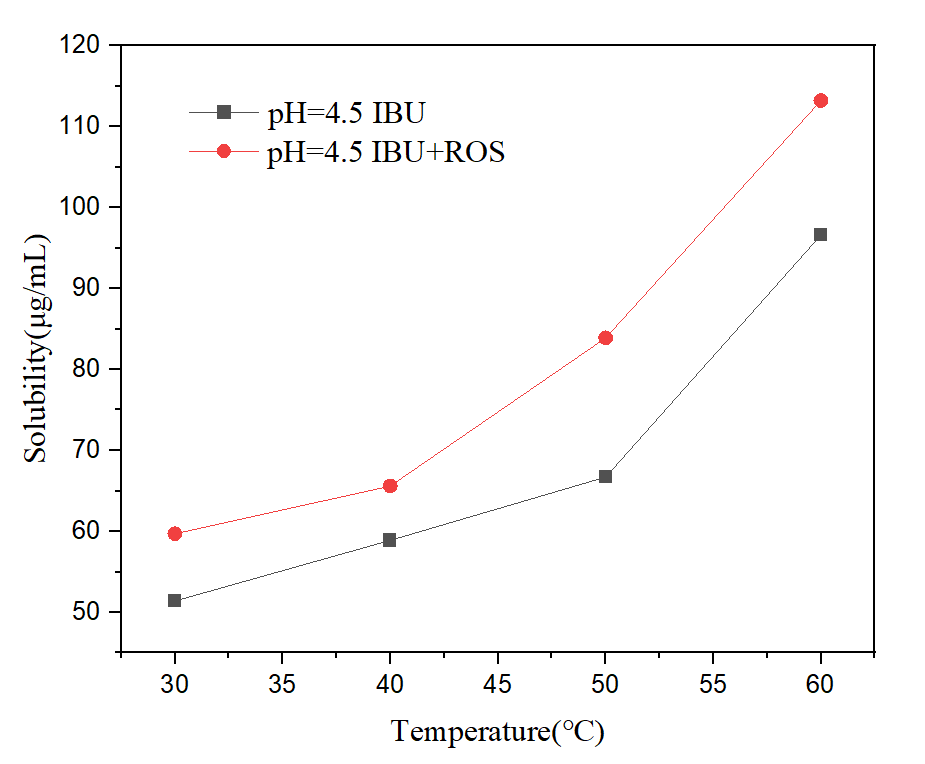


**Figure S6** Solubility temperature profiles: ibuprofen and ibuprofen containing rosiglitazone at pH 6.8.


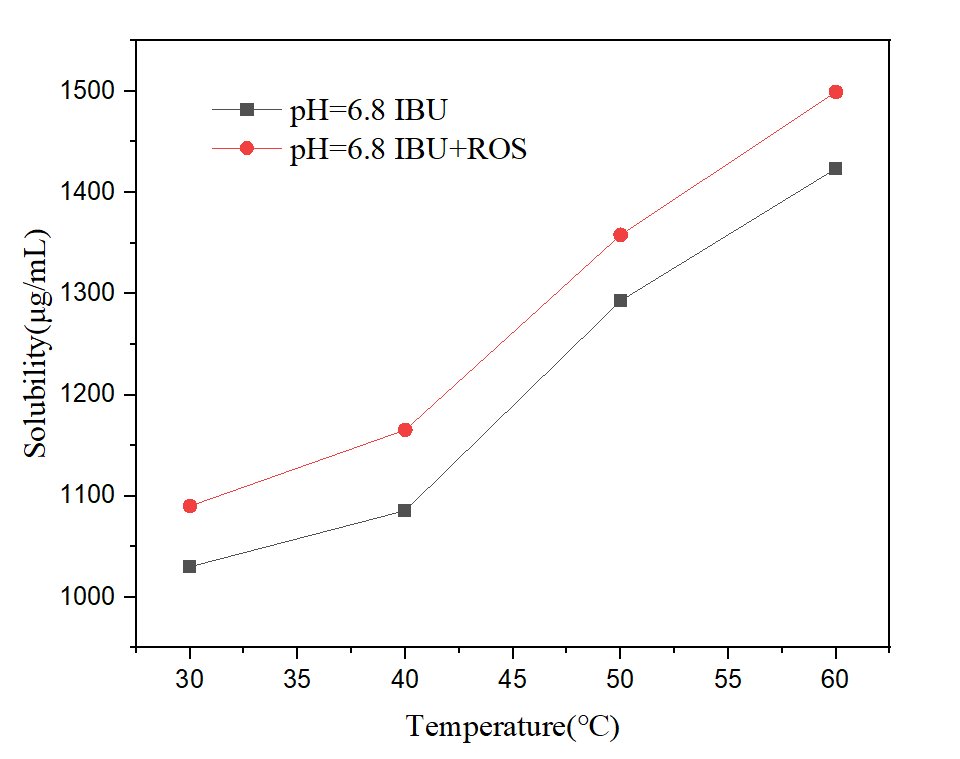

Supplement: S1 File — (DOCX) [file pone.0345185.s001.docx]
